# Breast cancer tumor microenvironment affects Treg/IL-17-producing Treg/Th17 cell axis: Molecular and therapeutic perspectives

**DOI:** 10.1016/j.omto.2023.01.001

**Published:** 2023-01-11

**Authors:** Farhad Seif, Zahra Torki, Hamidreza Zalpoor, Mehran Habibi, Majid Pornour

**Affiliations:** 1 Department of Immunology and Allergy, Academic Center for Education, Culture (ACECR), and Research (ACECR), Tehran, Iran; 2 Neuroscience Research Center, Iran University of Medical Sciences, Tehran, Iran; 3 Department of Medical Genetics, Faculty of Medicine, Tabriz University of Medical Sciences, Tabriz, Iran; 4 Shiraz Neuroscience Research Center, Shiraz University of Medical Sciences, Shiraz, Iran; 5 Network of Immunity in Infection, Malignancy, and Autoimmunity (NIIMA), Universal Scientific Education, and Research Network (USERN), Tehran, Iran; 6 Department of Surgery, Johns Hopkins Breast Center at Bayview Campus, Baltimore, MD, USA; 7 Department of Photo Healing and Regeneration, Medical Laser Research Center, Yara Institute, Academic Center for Education, Culture, and Research (ACECR), Tehran, Iran; 8 Department of Oncology, School of Medicine, University of Maryland, Baltimore, MD, USA

**Keywords:** breast cancer, tumor microenvironment, regulatory T cell, Treg, IL-17-producing Treg, Th17, microRNA, secretome, immune checkpoint inhibitor, cytokine

## Abstract

The tumor microenvironment (TME) comprises a variety of immune cells, among which T cells exert a prominent axial role in tumor development or anti-tumor responses in patients with breast cancer (BC). High or low levels of anti-inflammatory cytokines, such as transforming growth factor β, in the absence or presence of proinflammatory cytokines, such as interleukin-6 (IL-6), delineate the fate of T cells toward either regulatory T (Treg) or T helper 17 (Th17) cells, respectively. The transitional state of RORγt^+^Foxp3^+^ Treg (IL-17-producing Treg) resides in the middle of this reciprocal polarization, which is known as Treg/IL-17-producing Treg/Th17 cell axis. TME secretome, including microRNAs, cytokines, and extracellular vesicles, can significantly affect this axis. Furthermore, immune checkpoint inhibitors may be used to reconstruct immune cells; however, some of these novel therapies may favor tumor development. Therefore, understanding secretory and cell-associated factors involved in their differentiation or polarization and functions may be targeted for BC management. This review discusses microRNAs, cytokines, and extracellular vesicles (as secretome), as well as transcription factors and immune checkpoints (as cell-associated factors), which influence the Treg/IL-17-producing Treg/Th17 cell axis in BC. Furthermore, approved or ongoing clinical trials related to the modulation of this axis in the TME of BC are described to broaden new horizons of promising therapeutic approaches.

## Graphical abstract

**Figure:**
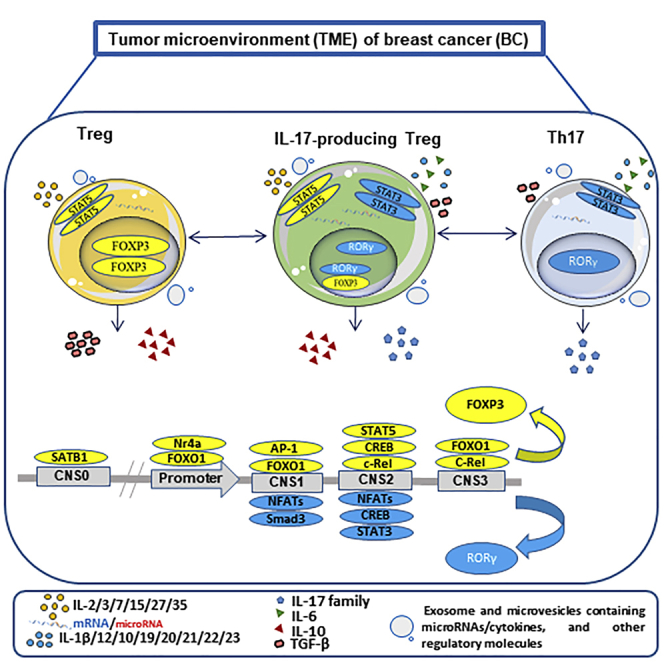

## Teaser

Tumor microenvironment (TME) affects the fate of immune cells, especially T cells, toward either inflammatory or regulatory phenotypes. TME contains microRNAs, cytokines, extracellular vesicles, transcription factors, and immune checkpoints that are potential therapeutic targets for the management of breast cancer, focusing on T cell polarization.

## Introduction

The tumor microenvironment (TME) provides an immunosuppressive condition that enables tumor cells to escape from the immune system by changing the function of innate and adaptive immune cells.^1^ Natural killer (NK) cells are one of the innate lymphoid cells that protect the healthy host cells by eradicating virus-infected, stressed, or tumor cells.^1^ In addition, cytotoxic T cells (CD8^+^ T cells) are the most prominent effector T cells that specifically recognize virus-infected or tumor cells. Naive CD4^+^ T helper (Th) cells commonly differentiate into several effector cells, including Th1, Th2, Th17, and regulatory T cells (Tregs).^2^^,^^3^ Recent successful cancer immunotherapies focus on adoptive cell transfer therapies with genetically modified receptors such as CARs to amplify and improve the function of CD8^+^ T cells.^4^ On the other hand, although immune checkpoints play pivotal roles in maintaining tolerance against autoimmune responses, they may exacerbate tumor development. Therefore, immune cells’ function may be restored through suppression of immune checkpoint inhibitors (either with monoclonal antibodies or other biological drugs).^4^ However, some of these novel therapies may favor tumor development. As a result, these therapeutics should be cautiously administered to achieve desirable responses.^5^

Since TME affects T cells, impaired functions and interactions of immune cells in TME potentially aggravate tumor development.^6^ TME induces immunosuppressive cells, including cancer-associated fibroblasts (CAFs),^7^ tumor-associated macrophages (TAMs),^8^ and myeloid-derived suppressor cells (MDSCs).^8^ Moreover, TME deviates tumor-killing T cells toward regulatory T cells (Tregs) and interleukin-17 (IL-17)-producing Tregs via TME secretome.^9^ Several studies have demonstrated that the secretome of TME, including microRNAs, cytokines, and extracellular vesicles, can prominently affect recruitment, differentiation, and polarization of Tregs through complicated crosstalks.^10^ MicroRNAs (miRNAs) are short (18–23 nt) non-coding RNAs that regulate different mRNAs post-transcriptionally. Dysregulation in miRNA expression may be evident in pathologic circumstances, for example, cancers. Recent studies have reported that miRNAs may affect tumor initiation and development, either as tumor-promoting miRNAs (oncomicroRNAs) or tumor-suppressor miRNAs.^9^ Tumor secretome can induce immune depletion by increasing Treg function and decreasing apoptosis in Tregs.^11^ Consequently, Tregs suppress the cytotoxic effects of T cells and NK cells by anti-inflammatory cytokines; meanwhile, they contribute to tumor development in TME. For example, they can induce angiogenesis by the same growth factor that induces apoptosis in immune cells.^10^

Recently, IL-17-producing Tregs have been identified to be associated with tumor invasiveness and poor disease prognosis; however, Th17 cells may play anti-tumor roles along with the production of IL-6 and IL-17 as inflammatory cytokines.^12^ IL-17, a proinflammatory cytokine, is associated with BC progression with regard to increasing survival, angiogenesis, and invasiveness of tumor cells.^12^ Transforming growth factor β (TGF-β) is an anti-inflammatory cytokine that is responsible for differentiation of CD4^+^ T cells into either Th17 or Treg cells, relying on the cytokine milieu. A high level of TGF-β in the absence of IL-6 drives Th17 cells toward RORγt^+^Foxp3^+^ Treg/Th17 cells. On the other hand, when IL-6 is higher than TGF-β, Foxp3^+^ Tregs can transdifferentiate into either Th17 or Treg/Th17 cells, respectively. This reciprocal transdifferentiation is known as the Treg/IL-17-producing Treg/Th17 cell axis.^13^ Impaired Treg/IL-17-producing Treg/Th17 cell axis has been reported in different kinds of cancers, especially breast cancer (BC).^9^ Therefore, this review discusses miRNAs, cytokines, and extracellular vesicles (as secretome) as well as transcription factors and immune checkpoints that influence the Treg/IL-17-producing Treg/Th17 cell axis in BC. Furthermore, approved or ongoing clinical trials related to the modulation of this axis in the TME of BC are presented as promising therapeutic approaches.

## Differentiation of Treg, IL-17-producing Treg, and Th17 cells in TME

**Figure 1:**
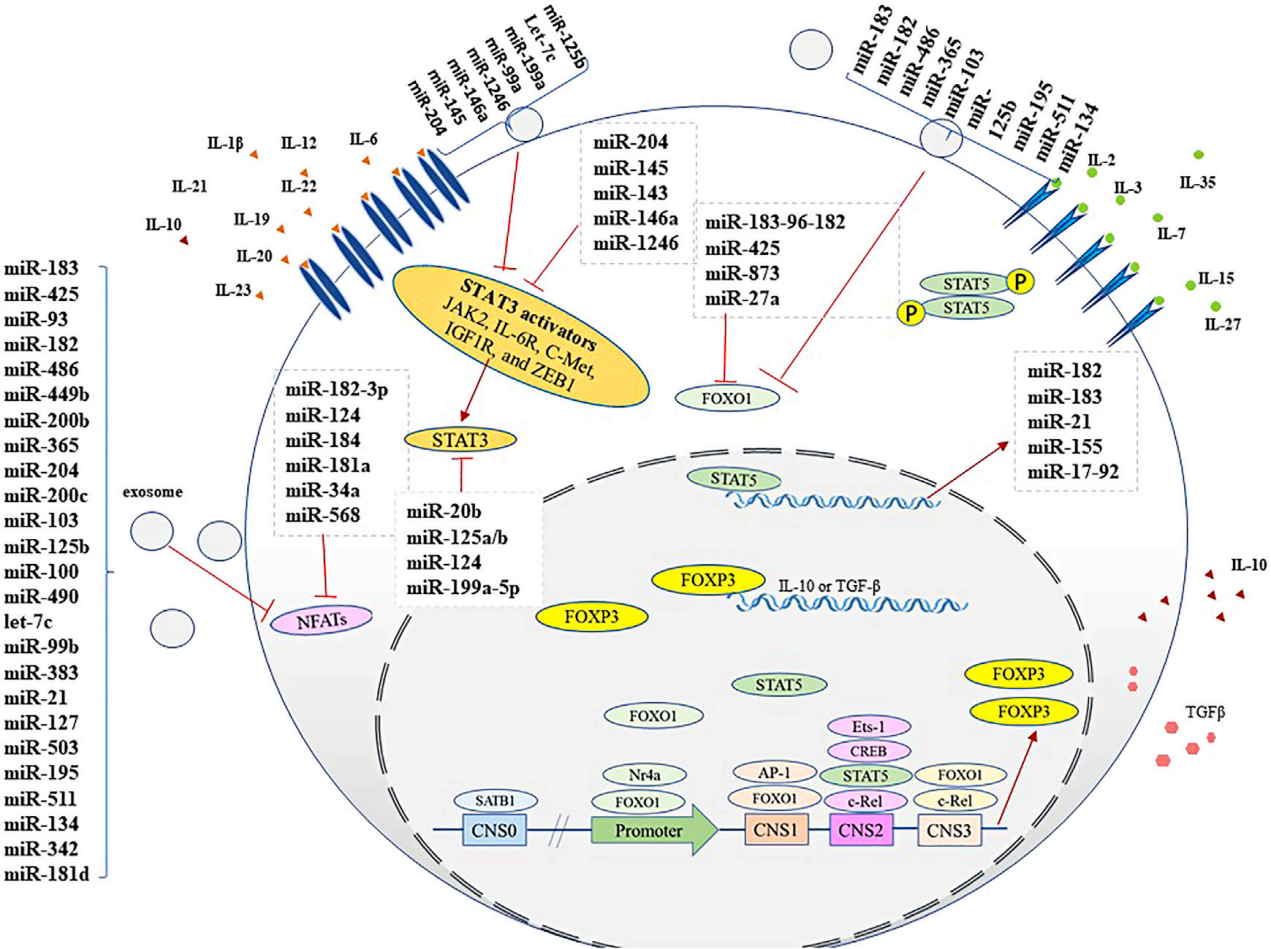
The importance of STAT5 and STAT3 balance in FOXP3 expression and Treg production in BC patients

At least two subtypes of Tregs have been observed in patients with BC based on their maturation site, including naturally CD4^+^CD25^+^ Tregs (nTregs) in the thymus and inducible Tregs (iTregs) in the peripheral tissues. Also, IL-17-producing Tregs are a transitional state between Tregs and Th17 cells.^13^ Although nTregs interact with the other effector and activate immune cells via cell-to-cell contact, iTregs represent such performance via the secretion of anti-inflammatory cytokines such as TGF-β and IL-10.^14^ Furthermore, IL-17-producing Tregs show their inhibitory effects by IL-10 secretion besides releasing some inflammatory cytokines such as IL-6 and IL-17 (Figure 1). A small population of CD4^+^CD25^+^FOXP3^+^ nTregs developed in the thymus has a vital role in preventing autoimmunity.^15^ iTregs have essential roles in protecting against chronic inflammatory conditions and likely play a key role in regulating immune responses to commensal microorganisms.^14^ Besides, iTregs have a role in peripheral tolerance and in preventing local inflammation when external antigens are present.^14^

### FOXP3 and RORγt

**Figure 2:**
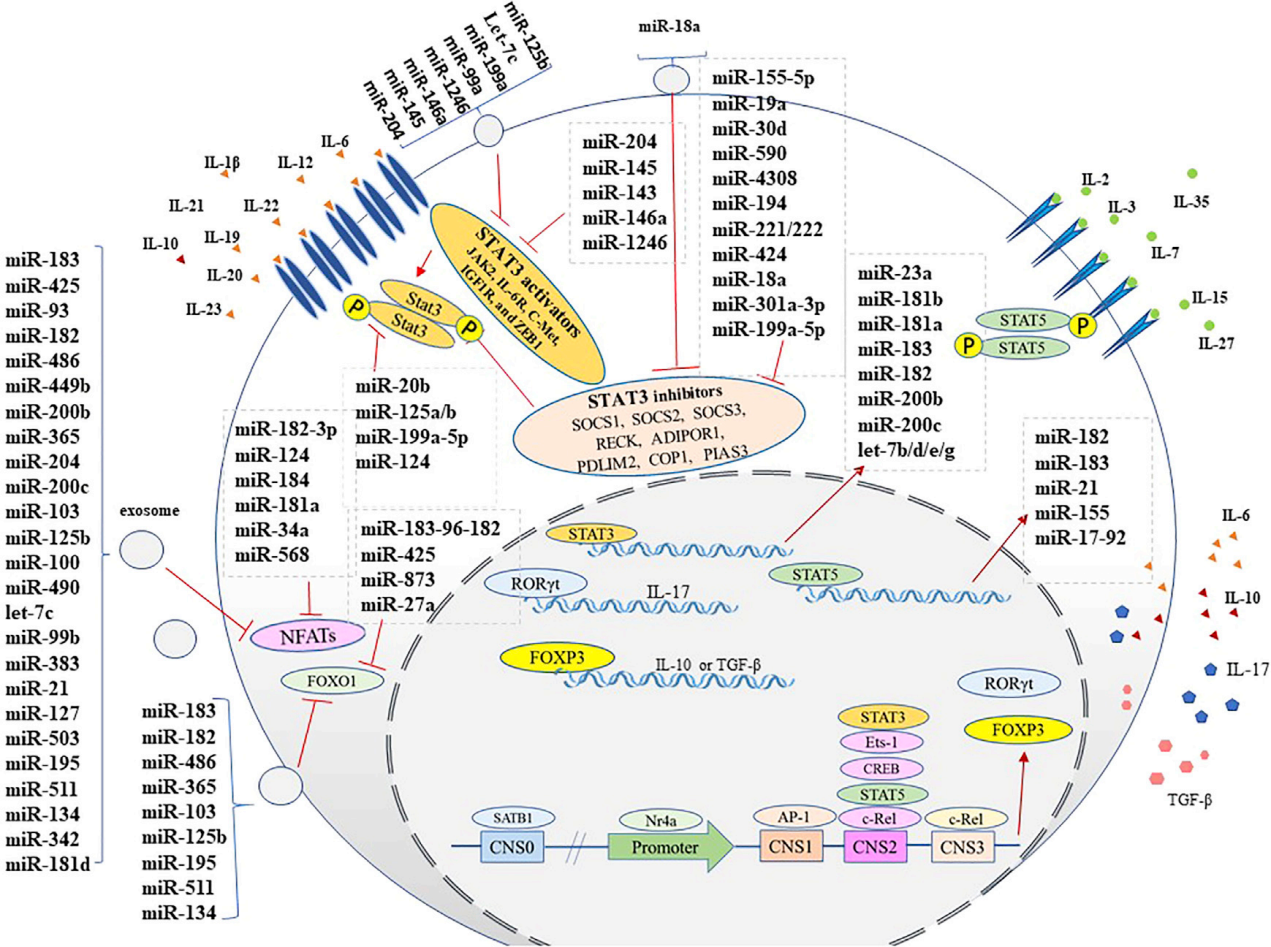
The effects of STAT3, NFATs, FOXO1, and STAT5 changes on FOXP3 reduction and IL-17-producing cells in BC patients

T cells need TGF-β along with IL-6 or IL-21 for converting to IL-17 producer cells, and differentiation of these cells into Th17 cells is dependent on the activation of receptor retinoic acid-related orphan receptor γt (RORγt).^15^ Th17 cells can secrete IL-17A, IL-17F, IL-21, and IL-22 cytokines utilized for neutrophil and macrophage recruitment and activation during inflammatory and autoimmune diseases.^15^ Forkhead box P3 (FOXP3) is a transcription factor required for Treg differentiation. Differentiation of both Treg and IL-17-producing Tregs needs the elevation of FOXP3 as a transcription factor.^16^ Thus, it seems that TGF-β can alter the balance of Treg and Th17 cells dependent on FOXP3 and RORγt frequency. It has been demonstrated that a combination of TGF-β and IL-6 is necessary for Th17 polarization, but TGF-β alone induced Treg polarization.^17^ However, a subpopulation of CD4^+^ FOXP3^+^ RORγt^+^ Treg cells represents the functional features of both Th17 and Treg cells.^9^^,^^17^ The promoter region of FOXP3, the hallmark transcription factor in Tregs, does not have permanent activity, and its function strongly depends on other *cis*-regulatory elements.^18^ Aside from the FOXP3 promoter, four conserved non-coding sequences (CNSs) serve as regulatory elements (*trans* elements) to which different transcription factors might bind and impact FOXP3 expression positively or negatively. For instance, the binding of c-Rel to CNS2 and CNS3 enhances FOXP3 induction, and activator protein-1 (AP-1) can transactivate FOXP3 expression by binding to CNS1.^18^ Furthermore, Foxp3^−^CD25^+^ Treg cell precursors in the thymus have detectable expression of Nr4a1 protein and the expression of nuclear receptor family members (or the Nr4a family of orphan nuclear receptors) Nr4a1, Nr4a2, and Nr4a3 is increased by T cell receptor (TCR) signaling, which leads to either redundant binding of Nr4a family members to the promoter of the FOXP3 gene (encoding FOXP3 that is a Treg-inducing transcription factor) or initiation of an adverse selection on Tregs (Figure 2).^18^

### FOXO1

The FOXO family has various effects on Treg fates and FOXP3 expression.^18^ SATB1, a Treg pioneer enhancer, binds to CNS0 and overexpresses FOXP3, and also induces cytotoxic T lymphocyte-associated antigen 4 (CTLA-4) and IL-2Ra expression as Treg differentiation-related genes.^18^ While reduction of FOXO1 balance against FOXP3 is necessary for Treg differentiation, FOXO1 and FOXO3 promote the expression of FOXP3 and Treg differentiation redundantly through attachment to CNS1 and CNS3 (Figure 1).^18^ CNS1, an enhancer in the upstream of the transcription start site of the promoter region of FOXP3, has conserved consensus-binding sites of FOXO1, and CNS3, which is located in the first intron of the FOXP3 gene, also is an enhancer of this gene.^19^ FOXO1 reduction may help the production of IL-17 by Tregs, which results in polarization of Tregs toward IL-17-producing Tregs.^9^ Smad family member 3 (Smad-3) and nuclear factor of activated T cells (NFAT) reduce FOXP3 expression via binding to CNS1, and NFAT forms a chromatin loop in the promoter region by attachment to CNS2 (Figure 2).^20^

### JAK-STAT pathway

FOXP3 binds to RORγt and ceases its transcriptional activity in the lack of IL-6 or IL-21.^21^ Hence, IL-6 or IL-21 helps Th17 differentiation by separation of RORγt from FOXP3. The Janus kinase (JAK) signal transducer and activator of transcription (STAT) pathway directs differentiation and polarization of T cells through some cytokine receptors.^2^^,^^22^ The maturation of FOXP3^+^ Tregs is mediated by a IL-2/IL-2R/STAT5-dependent process.^23^ In fact, STAT5 induces FOXP3 overexpression via IL-2 and Treg differentiation and reduces Th17 cell lineage.^16^ STAT3 can bind to the CNS2 region and FOXP3 downregulation, whereas STAT5, a transcription factor activated by IL-2, binds to CNS2 and helps maintain FOXP3 expression and stability of Tregs.^24^ STAT5 facilitates activation of the RAS/mitogen-activated protein kinase (MAPK)/ERK and PI3K/AKT through phosphorylation of key tyrosine residues in cytoplasmic IL-2R by JAK1 and JAK3 in the thymus. However, STAT5 helps FOXP3 transcription by binding to the promoter region of the CNS2 site.^23^ In contrast, other STATs, such as STAT4 and STAT6, inhibit FOXP3 expression.^25^ For example, IL-4 and IL-12 repress Treg progression by activating STAT6 and STAT4.^2^ IL-6 and IL-21 recruit STAT3, inducing dozens of miRNAs that induce IL-17-producing Tregs and Th17 cells.^9^ STAT5 drives activation of Tregs and induces Treg lineage commitment.^23^ Despite the inhibitory role of STAT3 on FOXP3 expression, it is indirectly involved in IL-17-producing Treg progression through release of RORγt from FOXP3.^21^ IL-23 can also stabilize Th17 differentiation via suppression of FOXP3 expression by activating both STAT3 and STAT4.^26^ Production of IL-17 can be inhibited by STAT5 and NCoR2 (a co-repressor protein), and IL-17-producing Tregs overexpress IL-17.^27^ This ability is related to IL-6-dependent STAT3 binding to enhancers within the IL-17 locus, and it competes with STAT5 and NCoR2 to bind this locus.^27^ Also, IL-2 and TGF-β reduce IL-6 receptor expression on iTregs. They prevent differentiation of Th17 cells,^28^ whereas IL-2 and IL-2R expression is diminished in IL-17-producing Tregs by elevating the rate of some miRNAs such as miR-182-183 cluster.^9^ Thus, IL-17-producing Treg or iTreg development in TME seems to be dependent on the balance of STAT3 and STAT5 signaling pathways. In the following, we describe the relationship of cytokines, miRNAs, and extracellular vesicles (as secretome), as well as transcription factors and immune checkpoint inhibition in the Treg/IL-17-producing Treg/Th17 cell axis in BC.

## The effect of secretome on Treg/IL-17-producing Treg/Th17 cell axis

### Cytokines

Several studies reported that tumor necrosis factor α (TNF-α), TGF-β, IL-1/IL-1β, IL-6, IL-7, IL-10, IL-13, IL-19, IL-20, colony-stimulating factor-1, IL-15, IL-17, IL-22, IL-23, and IL-35 are increased in concentrations in patients with BC.^29^ Also, it was observed that IL-2 has a dynamic variation in BC pathogenesis and affects the circulation of tumor cells in patients with advanced BC,^30^ while the level of IL-2 and the circulation of tumor cells have a reverse correlation in BC patients Although IL-2 can be used as monotherapy, its combination with some anti-cancer immunotherapies, e.g., antigen-specific vaccination, adoptive cell transfer, chimeric antigen receptor (CAR) T cell therapy, and immune checkpoint blockade has led to promising therapies.^31^ Some cytokines may affect Treg or IL-17-producing cells through phosphorylation of STAT3 or STAT5. Although IL-1β, IL-6, IL-10, IL-19, IL-20, IL-21, IL-22, and IL-23 activate STAT3 and suppress FOXP3 expression, IL-2, IL-3, IL-7, IL-15, IL-27, and IL-35 stimulate STAT5 and stabilize FOXP3 expression.^32^^,^^33^ Also, these cytokines may stimulate the expression of several miRNAs that control STAT3 activators or STAT3 modulators.^34^

Among cytokines in the BC TME, IL-2, IL-7, and IL-15 activate STAT5 and induce FOXP3 and Treg production. In contrast, IL-6, IL-17, and IL-21 activate STAT3 to IL-17 production and polarization of T cells toward Th17 and IL-17-producing Tregs.^35^^,^^36^ Even IL-10 induces STAT3 for its function in Tregs.^37^ Moreover, some cytokines, including IL-27 and IL-35, induce Treg production and constancy via STAT1 and STAT3.^33^ STAT5 drives activation of Tregs through overexpression of FOXP3, but STAT3 reduces FOXP3 and increases free RORγt levels and induces IL-17-producing T cells.^38^ Thus, as already mentioned, Treg and IL-17-producing Treg development may be affected by the balance of STAT3 and STAT5 signaling pathways. Even though IL-2 function is required for T cell activation, IL-2/IL-2RA signaling is impressive in Treg establishment. However, IL-2 and IL-2RA (CD25) expression in Tregs is very low.^9^ IL-2/IL-2RA has a prominent role in FOXP3 expression in Tregs.^24^ Nevertheless, different cytokines secreted in the TME of BC can activate STAT5 and STAT3. They may induce the expression of several miRNAs involved in Treg production and permanency.^35^^,^^36^^,^^37^ IL-2, IL-3, IL-7, IL-10, IL-15, IL-27, and IL-35 can induce Treg production, and most of them can activate STAT5.^37^^,^^39^ In contrast, IL-1β, IL-6, IL-21, IL-12, IL-19, IL-20, IL-22, and IL-23 repress the Treg polarization and increase IL-17-producer T cells. These cytokines induce IL-17 via activation of STAT3.^32^^,^^37^ However, IL-23, besides STAT3, persuaded STAT4 and stabilized only Th17.^40^ Both STAT3 and STAT5 are critical to expressing some miRNAs involved in Treg and IL-17-producing Treg polarization.^15^^,^^41^

### MicroRNAs in Treg/IL-17-producing Treg/Th17 cell axis modulation

FOXP3 transcription regulation enables the immune system to fine-tune Treg activity under various conditions.^42^ miRNAs can be involved in regulating the function and stability of Tregs in the TME. Uptake of tumor-derived elements such as miRNAs by Tregs may change the Treg future toward IL-17-producing T cells and alter the immune response substantially.^43^ Several miRNAs with different sources from TME cells, along with the miRNAs with intrinsic sources in T cells, influence the balance of the Treg/IL-17-producing Treg/Th17 cell axis (Table 1).^44^ Numerous miRNAs from endogenous or exogenous sources may be involved in the regulation of different types of Tregs and IL-17-producing T cells in BC. Invasive breast tumors had synchronic enrichment of Tregs, IL-17-producing Tregs, and Th17 cells associated with tumor aggressiveness. The induction of angiogenic factors by IL-17-producing T cells was related to the disease progression. Thus, disturbance of these miRNA profiles affects the fluctuation of the Treg/IL-17-producing Treg/Th17 cell axis in patients with BC.^9^

**Table 1:** MicroRNAs’ effects on the Th17/IL-17-producing/Treg cell axis in various signaling pathways in BC

miRNA	miRNA level in breast tumor and/or sera	Target genes	Related signaling pathway and/or transcription factor	Function in Treg/Th17/IL-17-producing Treg fate	References
miR-20b	breast tumor ↑ breast sera ↑	NFAT5, CAMTA1, STAT3, RORγt	NFAT, IL-6/STAT3, RORγt	Th17 ↓, Treg ↑	Xin et al. and Li et al.^45^^,^^46^
miR-182-3p	breast tumor ↑ breast sera ↑	IL-2RA, CD3d, ITK, NFATc3	IL-2/IL-2RA, TCR/CD3, NFAT	IL-17-producing Treg ↑^a^	Soheilifar et al.^9^
miR-182-5p	breast tumor ↑ breast sera ↑	FOXO1	FOXO1	IL-17-producing Treg ↑^a^	Soheilifar et al.^9^
miR-183C (miR-183-96-182 cluster)	breast tumor ↑ breast sera ↑	FOXO1	IL-6/STAT3, FOXO1	Th17 ↑, Treg ↓?	Soheilifar et al. and Ichiyama et al.^9^^,^^47^
miR-873	breast tumor ? breast sera ?	FOXO1	FOXO1	Th17 ↑, Treg ↓?	Liu et al.^48^
miR-425	breast tumor ↑ breast sera ↑	FOXO1	FOXO1	Th17 ↑, Treg ↓?	Zhang et al. and Itani et al.^49^^,^^50^
miR-590	breast tumor ↓ breast sera ?	Tob1, RECK	TCR/CD3, Akt/ERK, STAT3	Th17 ↑, Treg ↓?	Shen et al. and Zhou et al.^51^^,^^52^
miR-155	breast tumor ↑ breast sera ↑	SOCS1, SIRT1, Ets-1, IL-2ITK	IL-6//STAT3, TCR/CD3, IL-2/IL 2RA	Th17 ↑, Treg ↑, IL-17-producing Treg ↑^b^	Pasculli et al. and Zheng et al.^53^^,^^54^
miR-326	breast tumor ↓ breast sera ?	Ets-1	TCR/CD3	Th17 ↑, Treg ↓	Ghaemi et al. and Dolati et al.^55^^,^^56^
miR-125a	breast tumor ↓ breast sera ?	IL-6R, STAT3, Ets-1	IL-6/STAT3	Th17 ↓, Treg ↑	Yang et al. and Ge et al.^57^^,^^58^
MIR-125b	breast tumor (TNBC) ↑ breast sera ↑	STAT3	IL-6/STAT3	Th17↓, Treg ↑	Incoronato et al. and Tian et al.^59^^,^^60^
miR-124	breast tumor ↓ breast sera ?	SIRT1, NFATc1, PTBP1, CAMTA1, STAT3	IL-2/IL-2RA, NFAT, IL-6/STAT3	Th17 ↓, Treg ↑	Heyn et al. and Kang et al.^61^^,^^62^
miR-181c	breast tumor ↑ breast sera ↓	IL-2, Smad-7	IL-2/IL-2RA, TGF-β/Smad	Th17 ↑, Treg ↓?	Xue et al. and Zhang et al.^63^^,^^64^
miR-126	breast tumor ↓ breast sera ?	p85β	PI3K/Akt	Th17 ↓, Treg ↑	Qin et al. and Zou et al.^65^^,^^66^
miR-184	breast tumor ↓ breast sera ↓	NFATc2	NFAT	Th17 ↓?, Treg ↑?	Weitzel et al. and Fu et al.^67^^,^^68^
miR-181a	breast tumor ↓/↑ breast sera ↓	NFAT5	PI3K/Akt, NFAT	Th17 ↓, Treg ↑	Łyszkiewicz et al. and Serr et al.^69^^,^^70^
miR-34a	breast tumor ↓ breast sera ↑	PLCG1, CD3E, PIK3CB, TAB2, NFΚBIA, NFATc4	TCR/CD3, PI3K/Akt, NF-κB, NFAT	Th17 ↑, Treg ↓	Hart et al. and Diener et al.^71^^,^^72^
miR-202-3p	breast tumor ↓ breast sera ?	CD3 zeta (CD247)	TCR/CD3	Th17 ↑?, Treg ↓?	Fornari et al. and Gao et al.^73^^,^^74^
miR-31	breast tumor (basal like BC) ↑ breast sera ↑	Gprc5a	TCR/CD3	Th17 ↑?, Treg ↓	Moffett et al. and Lu et al.^75^^,^^76^
miR-451	breast tumor ↓ breast sera ?	–	TCR/CD3	Th17 ↑, Treg ↓?	Liu et al. and Du et al.^77^^,^^78^
miR-146a	breast tumor ↑ breast sera ↑	TRAF6, IRAK1	TCR/CD3, NF-κB	Th17 ↓, Treg ↑	He et al. and Li et al.^79^^,^^80^
miR-20a	breast tumor ↑ breast sera ↑	MAP3K9	MAPK	Th17 ↑?, Treg ↓	Schwarzenbach et al. and Wang et al.^81^^,^^82^
miR-19b	breast tumor ↑ breast sera ↑	PTEN	PI3K/Akt/mTOR	Th17 ↑, Treg ↓	Liu et al. and Zou et al.^83^^,^^84^
miR-214	breast tumor (TNBC) ↑ breast sera ↑	PTEN	PI3K/Akt/mTOR	Th17 ↓, Treg ↑	Ahmadian-Elmi et al. and Zhu et al.^85^^,^^86^
miR-520b	breast tumor ↑ breast sera ?	PTEN	PI3K/Akt/mTOR	Th17 ↓?, Treg ↑	Zhu et al. and Zhang et al.^87^^,^^88^
miR-99a	breast tumor ↓ breast sera ↓	mTOR	PI3K/Akt/mTOR	Th17 ↓, Treg ↑	Warth et al. and Qin et al.^89^^,^^90^
miR-150	breast tumor ↑ breast sera ?	mTOR	PI3K/Akt/mTOR	Th17 ↓, Treg ↑	Warth et al. and Huang et al.^89^^,^^91^
miR-15b/miR-16	breast tumor ? breast sera ?	mTORC2 component Rictor	PI3K/Akt/mTOR	Th17 ↓, Treg ↑	Singh et al. and Liu et al.^92^^,^^93^
miR-10a	breast tumor ↓ breast sera ?	Bcl-6, Ncor2	PI3K/Akt/mTOR, Bcl-6	Th17 ↓, Treg ↑	Jeker et al. and Zhang et al.^39^^,^^94^
miR-10a-3p	breast tumor ? breast sera ?	REG3A	JAK2/STAT3	Th17 ↓, Treg ↑	You et al.^95^
miR-19a	breast tumor ↓ breast sera ↑	SOCS1	IL-6/STAT3	Th17 ↑?, Treg ↓?	Sochor et al. and Lin et al.^96^^,^^97^
miR-30d	breast tumor ↓ breast sera ?	SOCS1	IL-6/STAT3	Th17 ↑?, Treg ↓?	Kobayashi et al. and Croset et al.^98^^,^^99^
miR424	breast tumor ↑ breast sera ↑	COP1	STAT3	Th17 ↑, Treg ↓	Dallavalle et al. and Ding et al.^100^^,^^101^
miR-221	breast tumor ↑ breast sera ↑	SOCS3, IRF2	JAK/STAT	Th17 ↑?, Treg ↓?	Kneitz et al. and Li et al.^102^^,^^103^
miR-199a-5p	breast tumor ↓ breast sera ?	PIAS3, STAT3, Ets-1	STAT3, Akt/mTOR	Th17 ↓, Treg ↑?	Wang et al. and Li et al.^104^^,^^105^
miR-139-5p	breast tumor ↓ breast sera ?	Rap1b, NF-κB	MAPK, NF-κB, STAT3	Th17 ↑?, Treg ↓?	Zou et al. and Haakensen et al.^106^^,^^107^
miR-222-3p	breast tumor ↑ breast sera ↑	SOCS3	IL-6/STAT3	Th17 ↑?, Treg ↓?	Ying et al. and Ahmed et al.^108^^,^^109^
miR-4308	breast tumor ? breast sera ?	SOCS3	IL-6/STAT3	Th17 ↑?, Treg ↓?	Wang et al.^110^
miR-203	breast tumor ↑ breast sera ↑	SOCS3	IL-6/STAT3	Th17 ↑?, Treg ↓?	Muhammad et al. and Madhavan et al.^111^^,^^112^
miR-18a	breast tumor ↑ breast sera ↑	PIAS3, Smad4, HIF-1α, Rora	IL-6/STAT3, TGF-β	Th17 ↓, Treg ↑	Montoya et al. and Nair et al.^113^^,^^114^
miR-210	breast tumor ↑ breast sera (HER2-positive breast sera) ↑	HIF-1α	HIF-1α	Th17 ↑ ↓, Treg ↓	Chen et al. and Wu et al.^115^^,^^116^
miR-221/-222	breast tumor ↑ breast sera ↑	PDLIM2, IL-23R, MAF	IL-6/STAT3, IL-23	Th17 ↓, Treg ↑?	Liu et al. and Mikami et al.^117^^,^^118^
miR-212	breast tumor ↓ breast sera ?	Bcl-6	Bcl-6	Th17 ↑, Treg ↓?	Nakahama et al. and Damavandi et al.^119^^,^^120^
miR-30a	breast tumor ↓ breast sera ?	IL-21R	IL-21	Th17 ↓, Treg ↑?	Qu et al. and Miao et al.^121^^,^^122^
miR-26a	breast tumor ↓ breast sera ↓	IL-6	IL-6	Th17 ↓, Treg ↑	Zhang et al. and Huang et al.^123^^,^^124^
miR-98-5p	breast tumor ? breast sera ?	IL-6	IL-6	Th17 ↓, Treg ↑	Xu et al.^125^
miR-301a-3p	breast tumor ↑ breast sera ?	PIAS3	STAT3	Th17 ↑, Treg ↓?	Tang et al.^126^
miR-21	breast tumor ↑ breast sera ↑	SATB1, Smad-7	TGF-β/Smad	Th17 ↑, Treg ↑↓	Murugaiyan et al., Yan et al., and Liu et al.^127^^,^^128^^,^^129^

?, needs further confirmation.

↑? or ↓?, speculated increased or decreased differentiation of the Treg or Th17 cells, respectively, without functional proof.

a Presence all three populations including Th17, Treg IL-17-producing Treg was technically confirmed.

b Presence of both Th17 and Treg populations was technically confirmed as well as IL-17-producing Treg possibility seems to be inevitable and needs further confirmation.

#### MicroRNAs that positively and negatively affect Treg/IL-17-producing Treg/Th17 cell axis

Considering the observed contents, overexpression and stability of FOXP3 play an essential role in the differentiation of T cells toward Tregs and their constancy.^42^ Therefore, miRNAs that modulate FOXP3 and impair its inhibitors and activators can be effective in the fate of Tregs.^42^ miR-155, miR-146a, miR-21, miR-95, miR-7, miR-34a, miR-18a, and let-7d are effective in the generation of Tregs and their function. miR-21 is overexpressed in Tregs and positively controls FOXP3 expression, and Treg stability and proliferation.^130^ miR-146a or let-7d, miR-7, miR-18a, miR-34a, and miR-155 cooperate to gain inhibitory properties in Tregs. For instance, miR-146a suppresses the interferon-γ (IFN-γ)/STAT1 pathways by targeting the expression of STAT1 and promotes Treg-suppressive function.^130^ SATB1, a protein involved in chromatin assembly and expression of different genes, is required to prevent Th cell polarization, but it is suppressed directly by FOXP3 activation after maturation of Tregs.^130^ miR-155, miR-21, miR-7, miR-34a, and miR-18a target SATB1 and help stabilize immune inhibitory functions of Tregs. miR-15a contributes to Treg-mediated suppression of optimal maturation of dendritic cells through impairing CTLA-4 expression in Tregs. The exosomes derived from Tregs include suppressive miRNAs such as let-7d, which can cease the proliferation of Th1 and the secretion of Th1-relevant cytokines *in vivo*.^130^ Survival and proliferation of Tregs can increase by elevation of IL-2 sensitivity through miR-155 enhancement.^61^^,^^131^ Despite the inhibitory effects of miR-17-miR-92 on FOXP3 expression in Tregs, low-rate presence of miR-17-miR-92 is necessary to prevent apoptosis in Tregs.^130^

Some miRNAs are negative regulators for differentiation of iTregs, consisting of miR-31, miR-24, miR-210, miR-17-miR-92 cluster, miR-15a, and miR-149-3p.^132^^,^^133^ It has been reported that these miRNAs impair the formation of iTregs by targeting FOXP3 and reducing their immune inhibitory effects. Moreover, miR-15a decreases CTLA-4 expression along with FOXP3, and the miR-23-miR-27-miR-24 cluster suppresses TGF-β-mediated pathways involved in Treg induction.^130^^,^^132^ Detailed investigation related to the profile of miRNAs circulating in sera of patients with BC rarely confirmed the elevation of these types of miRNAs in the TME secretome.^9^

#### Endogenous and exogenous microRNAs involved in Treg/IL-17-producing Treg/Th17 cell axis

During carcinogenesis, the profile of miRNAs in T cells is converted to modify the normal state of the niche to a tumor-favorable condition and contributes to the polarization of T cells into different kinds of Tregs, IL-17-producing Tregs, and Th17 cells.^9^^,^^134^ It is a balance between Treg and Th17 cells that is convertible depending on the activation of different signaling pathways such as STAT5, STAT3, TCR, and Smad.^38^^,^^134^ A significant upregulation of miRNAs has been demonstrated in CD4^+^ Th cells or Th17 cells.^83^^,^^85^^,^^119^^,^^127^^,^^135^^,^^136^^,^^137^ In contrast, downregulation of some miRNAs has been observed in these cells.^85^^,^^121^^,^^123^^,^^138^ On the other hand, the effects of several miRNAs have also been shown on different kinds of Tregs (Table 1).^9^^,^^45^^,^^47^^,^^61^^,^^67^^,^^69^^,^^89^^,^^92^^,^^139^ Different isoforms of miRNAs such as miR-182 and miR-183 can be simultaneously involved in the differentiation of both Treg and IL-17-producing T cells.^9^^,^^47^

Tumor cells and the other cells in the TME, especially immune inhibitory cells such as CAFs, TAMs, and MDSCs, affect other cells by secreting some vehicles such as microvesicle and exosome shuttled regulatory aspects, particularly miRNAs, and influence the fates of other cells.^140^ Tumor cells also transfer some oncomicroRNAs via these vehicles, affect the gene expression profile of target cells, especially T cells, and modify them toward inhibitory cells.^140^ Different studies have shown that dozens of oncomicroRNAs have been elevated in tumor tissue simultaneously and circulate in the sera of patients with BC.^141^ Profiling of these miRNAs reveal that most of them influence the expression of different targets in various signal transduction pathways involved in short-lived effector T cells. Their presence impacts the fate of T cells toward iTreg and IL-17-producing T cells.^9^ Moreover, they modulate IL-6, IL-17, IL-10, and TGF-β production. They often impact IL-10 and TGF-β secretion in tumor tissue to show inhibitory functions.^142^ Each of these cytokines affects BC and Treg/IL-17-producing Treg/Th17 cell fates; for example, T cells need to TGF-β along with IL-6 or IL-21 for conversion to IL-17-producing cells, and differentiation of these cells is related to activation of RORγt.^143^ Activation of IL-6-dependent STAT3 reduces the expression of STAT5 and induces RORγt in Th17 and T17/Tregs.^144^ While iTregs represent immune modulatory effects via secretion of anti-inflammatory cytokines, such as TGF-β and IL-10, IL-10 induces Tregs for secretion of considerable levels of IFN-γ, TGF-β, and IL-5 upon T cell receptor-mediated activation. On the other hand, IL-10 has been shown to be associated with cell proliferation and metastasis in BC.^145^

Several high-throughput studies demonstrated the overexpression of oncomicroRNAs and overcirculation in tumors, sera, and peripheral blood mononuclear cells (PBMCs) of patients with BC.^61^^,^^141^ For instance, increases in miR-182-5p, miR-182-3p, miR-183, miR-181b-1, miR-150, miR-21, and miR-103 levels were confirmed in tumors, sera, and PBMCs of patients with BC.^9^^,^^91^^,^^96^^,^^128^^,^^146^ Higher levels of miR-125b and miR-200c in plasma and tumors of triple-negative and non-invasive BC samples have been shown.^59^^,^^147^^,^^148^ These miRNAs impose immunosuppressive effects on T cells and target the transcripts of some critical proteins involved in the TCR/CD3 complex-associated signaling pathway, NFAT, and FOXO1. Altogether, these proteins may control FOXP3 fluctuation and free RORα/γ levels in CD4^+^ Th cells. Consequently, they stimulate iTregs, IL-17-producing Tregs, and Th17 polarization in patients with BC.^9^

#### FOXP3 and RORγt regulating microRNAs

Both IL-2 and IL-6 induce miR-182-183 cluster expression via STAT5 and STAT3 activation.^9^ Although STAT5 induces FOXP3 overexpression, STAT3 reduces FOXP3 expression and leads to separation of RORγt from FOXP3 and, subsequently, Th17 production as well as IL-17 secretion.^38^ However, FOXO1 affects FOXP3 and reduces NFATs by miR-182-183 cluster. Furthermore, other miRNAs with similar effects impact FOXP3 overexpression, IL-17, and TGF-β production by IL-17-producing Tregs.^9^^,^^38^ It seems that the presence of IL-17-producing Tregs may be a transitional situation between Tregs and Th17 as a reason for driving different signaling pathways with opposite effects, such as STAT3 and STAT5 in patients with BC.^149^ Most of the miRNAs summarized in Table 1 have a similar opposite potential in Treg/Th17/IL-17-producing Treg polarization. miR-183C (miR-183-96-182 cluster), miR-873, and miR-425 target FOXO1 and facilitate the differentiation of IL-17-producing T cells.^9^^,^^41^^,^^47^^,^^48^^,^^150^ It has been observed that miR-20b strongly downregulates RORγ, thereby inhibiting Th17 cell differentiation as well as affecting Treg production and maintenance.^138^^,^^149^ Overexpression of miR-182-183 cluster targets NFAT proteins such as FOXP3 suppressors and induces Tregs. However, increasing the miR-182-183 cluster reduces FOXO1 and consequently induces IL-17 production in FOXP3^+^ T cells.^9^

#### NFAT regulating microRNAs

NFAT families play a pivotal role in Th1 and Th2 differentiation and activation through TCR signaling.^151^ However, miR-182-5p, miR-182-3p, and miR-183 inhibit different kinds of NFATs and cease the differentiation of T cells toward Th1 and Th2 cells.^9^^,^^151^ For example, miR-184 suppresses NFAT1 (NFATc2) production, and miR-181 downregulates different kinds of NFAT.^67^^,^^70^ miR-182 and miR-183 were elevated in PBMCs of patients with BC and overcirculated in their sera. They reduced translation of NFATc2, NFATc3, and NFATc4 and suppressed Th1 differentiation in patients with BC.^9^ Also, miR-204 can target NFAT1.^152^ Besides the aforementioned miRNAs, most of the miRNAs mentioned in Table 1 can target different kinds of NFAT proteins. Also, they circulate at higher levels in the sera of patients with BC.^9^ Higher levels of miR-200c-suppressed NFATc1-Sox2 complex were reported in the serum of estrogen receptor positive and progesterone receptor positive BC patients.^147^^,^^153^ Calcineurin upregulates the activation of NFAT transcription factor family with calmodulin-dependent phosphatase activity. After TCR activation, calcineurin dephosphorylates NFATs to facilitate their nuclear translocation.^139^

#### B cell lymphoma 6 regulating microRNAs

B cell lymphoma 6 (Bcl-6) promotes Treg progression and is an inhibitor for Th17 polarization.^119^ The activation of co-stimulatory receptors such as ICOS, CD28, and STAT3 inducer cytokines such as IL-6 and IL-21 stimulates the PI3K-induced Bcl-6. However, STAT5 activation can indirectly stimulate Bcl-6 inhibitors such as T-bet and Blimp, subsequently leading to its suppression. Since IL-2 concentration modulates the balance between Bcl-6 and T-bet, a low-IL-2 condition positively affects Bcl-6 expression. Thus, it is never far from the mind that Bcl-6 is activated only at low levels of IL-2, while high concentrations of IL-2 cease Bcl-6. This can be used as a compensation mechanism for the loss of IL-2 in Treg polarization.^154^ Increasing Bcl-6 inhibits the expression of Treg suppressor miRNAs, including miR-17-92, miR-142-3p/-5p, and miR-31, whereas Bcl-6 targeting miRNAs drive polarization of IL-17-producing cells.^154^^,^^155^^,^^156^^,^^157^ miR-212 and miR-10a target Bcl-6 and induce Th17 production.^119^^,^^154^

#### mTOR and PTEN regulating microRNAs

The mammalian target of rapamycin (mTOR) signaling function diminishes the possibility of human iTreg generation by reducing FOXP3 expression.^83^^,^^92^ Also, IL-1β and IL-6 cooperation reduce FOXP3 expression during IL-17-producing T cell polarization through induction of MAPK and the Akt/mTOR pathway. In addition, this function is required for Th17 maintenance.^44^ Phosphorylated STAT3 promotes overexpression of RORγt and its separation from FOXP3.^38^ Phosphatase and tensin homology (PTEN), as a PI3K/AKT/mTOR inhibitor, reduces Th1 differentiation and helps Treg stability. miR-19b targets the transcripts of PTEN and enhances IL-17-producing T cell polarization.^83^ By contrast, tumor-cell-secreted miR-214 targets PTEN, which could be regarded as a switch that induces Treg expansion.^158^ Some miRNAs such as miR-99a and miR-150 suppress mTOR signaling and increase the differentiation of Tregs. Transcripts of mTOR are targeted by miR-15b and miR-16 and induce FOXP3 expression.^89^^,^^92^ However, among the miRs that affect mTOR signaling related factors, only a higher circulation of miR-16 was confirmed in BC patients’ sera.^81^

#### TCR/CD3 regulating microRNAs

TCR/CD3 signaling inhibition attenuates T cell activation and induces the formation of CD4^+^FOPXP3^+^ Tregs.^9^ Interaction between programmed cell death protein 1 (PD-1) and PD-2 on the surface of T cells and its ligand PD-L1 on the surface of tumor cells or APCs can drive T cell fates toward Tregs through ZAP70 inhibition by SHP2 and attenuates all three classic T cell activating pathways, namely MAPK, inositol 1,4,5-trisphosphate (Insp3), and diacylglycerol (DAG).^159^^,^^160^ Also, the TME secretome attenuates T cell activation through the prevention of expression of various TCR/CD3 chains and some adaptor proteins such as inducible T cell kinase (ITK).^9^ miR-182 and miR-34a can target CD3d and CD3e, respectively. In addition, miR-182 reduces ITK.^9^^,^^71^ Targeting CD3 ζ chain (CD247) by miR-202-3p has been shown in an *in vivo* study.^73^ Also, miR-31 and miR-451 reduce CD4^+^ and CD8^+^ T cell functions.^44^^,^^149^ For instance, co-stimulation of CD28 and TCR induces the expression of miR-31, which has an important role in CD8^+^ T cell exhaustion.^75^^,^^161^ miR-146a targets TRAF6, IRAK1, and nuclear factor κB (NF-κB) signaling, as well as suppressing TCR signaling in CD4^+^ Th cells.^44^,^79^ miR-20a can target MAP3K9, and the knockdown of this miRNA enhances Treg differentiation.^82^ Moreover, miR-155, which targets ITK, has higher circulation in sera of patients with BC.^162^

#### STAT5/STAT3 regulating microRNAs

STAT5 induces the expression of miR-182, miR-183, miR-21, and miR-155, which are positively involved in Treg and IL-17-producing Treg differentiation.^9^^,^^17^^,^^163^^,^^164^^,^^165^ Furthermore, STAT5 controls the negative regulators of Treg production, such as the miR-17-92 cluster, which targets FOXP3.^166^ Despite the inhibitory effect of STAT3 on FOXP3 expression, STAT3 induces the expression of miR-23a, miR-21, miR-181b-1, Let-7b/day/e/g, miR-181a, miR-183, miR-182, miR-200b, miR-200c, all of which are involved in both Treg and iTreg production, function, and stability.^9^^,^^47^^,^^164^^,^^167^ Also, miR-20b targets STAT3 directly and suppresses Th17 differentiation.^138^ In contrast, miR-141 targets STAT5a and induces Th17 differentiation.^137^ Moreover, miR-204, miR-1246, miR-145, miR-125a/b, and miR-146a, which are overexpressed or overcirculated in the TME of BC, target the proteins involved in STAT3 pathways, such as JAK2, IL-6R, C-Met, IGF1R, and ZEB1.^9^^,^^59^^,^^60^^,^^129^^,^^152^^,^^168^^,^^169^^,^^170^ miR-18a and miR-301a-3p target protein inhibitors of activated STAT3 (PIAS3) and induce the polarization of Th17 cells.^126^^,^^171^ In contrast, miR-18a has been identified as an inhibitor of Th17 differentiation by regulating Smad4, HIF-1α, and RORα.^113^

Some miRNAs, which target signaling pathways of IL-21 and IL-6 receptors, inhibit Th17 differentiation.^41^^,^^172^ It has been reported that miR-30a targets IL-21R. Also, miR-26a and miR-98-5p inhibit IL-6.^121^^,^^123^^,^^125^ Moreover, miR-19a-3p binds to the 3′ UTR of Fra-1 and limits its expression as well as the activation of its downstream pathway (IL-6/JAK/STAT3).^173^ The inhibitors of STAT3 signaling include suppressors of cytokine signaling 1 (SOCS1), SOCS2, SOCS3, RECK, ADIPOR1, PDLIM2, and COP1.^164^ SOCS1, an IL-6-STAT3 signaling suppressor, can be targeted by miR-155-5p, miR-19a, and miR-30d. Moreover, SOCS2 is diminished by miR-194.^98^^,^^174^^,^^175^ SOCS3 can be targeted by miR-384, miR-221, miR-222-3p, miR-4308, miR-203, and miR-206. These miRNAs affect the balance of Treg/IL-17-producing Treg/Th17 toward Th17 and IL-17-producing Treg cells.^102^^,^^108^^,^^110^^,^^111^^,^^135^^,^^176^ Also, miR-590-5p, miR-424, and miR-221/-222 target RECK, COP1, and PDLIM2, and miR-221/-222 reduces ADIPOR1.^51^^,^^100^^,^^117^^,^^177^ miR-199a-5p, miR-205, miR-373, and miR-139-5p induce STAT3 signaling by unknown mechanisms and induce Th17 and IL-17-producing Treg polarization,^104^^,^^106^^,^^178^ although it was reported that miR-199a-5p controls PIAS3 as a negative regulator of STAT3. In contrast, it was observed that miR-199a-5p directly downregulates STAT3 and suppresses Th17 differentiation.^104^^,^^105^

It is worth noting that upregulation and overcirculation of miR-181b, miR-183, miR-182-3p, miR-21, miR-18a-3p, miR-155-5p, miR-19a, and miR-221/-222 have been shown in tumor tissue and sera of BC patients.^9^^,^^53^^,^^96^^,^^162^^,^^179^^,^^180^^,^^181^^,^^182^^,^^183^^,^^184^^,^^185^ Therefore, the TME secretome of BC possibly takes part in Treg production and maintenance through the higher circulation of miRNAs which are STAT3 inhibitors, such as miR-125b and miR-145.^60^^,^^129^^,^^169^ A similar effect associated with the TME secretome of BC regarding IL-17-producing Treg polarization is inevitable by overcirculation of miR-182-5p/-3p, miR-21, miR-181b, miR-155-5p, and let-7a (Figure 2).^9^^,^^96^^,^^127^^,^^129^^,^^171^^,^^186^^,^^187^^,^^188^ Thus, it seems that tumor secretome and TME components, including cytokines and transferring vehicles (such as exosomes) in BC, force T cells to acquire Treg or IL-17-producing Treg characteristics in an orchestral manner. Both JAK/STAT and calcineurin/NFAT are FOXP3 expression-controlling signaling cascades in T cells.^189^ STAT3 and STAT5 bind to a CNS in the *FOXP3* promoter in tumor-infiltrating Tregs. STAT5 increases FOXP3 expression in Tregs, while STAT3 is a key transcription factor for Th17 differentiation.^190^ STAT3, as an anti-cancer target, may exert immune activation rescue in the TME.^191^ The transducer of ERBB2-1 (Tob-1) can suppress IL-2 production in Th17 cells and can be targeted by miR-590.^136^ E26 transformation-specific-1 (Ets-1), a protein in combination with STAT5, impairs IL-17 production and is targeted by miR-155, miR-141, and miR-326, and these miRNAs enhance IL-17 production.^192^^,^^193^ In contrast, miR-155 and miR-124a influence the expression of FOXP3 and iTreg generation positively through inhibition of histone deacetylase sirtuin 1 (SIRT1). Moreover, miR-155 induces Treg polarization by reduction of higher suppressor of cytokine signaling 1 (SOCS1), which is the main inhibitor of STAT5 in the IL-2 signaling pathway.^61^^,^^130^^,^^131^ IL-2 can stimulate several miRNAs involved in Treg polarization, such as miR-182-183 cluster, by activation of STAT5. However, the miR-182-183 cluster controls IL-2/IL-2RA signaling by directly targeting IL-2RA and regulating IL-2 expression.^9^ miR-182-183 can diminish IL-2 expression indirectly via reduction of NFATc4.^9^ Also, miR-181c-5p targeted the 3′ UTR of IL-2 mRNA, miR-568, and miR-20b, and miR-124 suppressed IL-2 expression via NFAT5 targeting.^45^^,^^62^^,^^63^^,^^130^^,^^139^ Furthermore, higher levels of miR-126 promote Treg stimulation; subsequently, it induces IL-10 expression.^65^

## Clinical perspective regarding Treg/IL-17-producing Treg/Th17 cell axis modulation in BC cancer

In the TME, secretory components, including cytokines and cytokine receptors, exosomes containing miRNAs and proteins, together with their cellular composition, have the capacity to shape the tumor and the immune cell polarization toward tumor tolerance;^194^ thus, targeting the secretome may be helpful in suppression of this phenomenon. The focus of cancer immunotherapy has shifted from the tumor to the TME, making it the new pillar of cancer treatment. Numerous immunotherapy strategies have shown effectiveness in developing long-term therapeutic responses.^195^ Manipulating non-cancerous components of the BC TME as well as targeting the T cell regulatory pathways and immune checkpoint molecules,^196^ in addition to correcting miRNA deregulation using miRNA-based therapeutics, extracellular vesicle formation and release, and indoleamine 2,3-dioxygenase (IDO) expression have been proposed in several research studies.^197^ Given that BC is a complex and heterogeneous disease, here we address various anti-cancer and immunotherapeutic agents in preclinical and clinical contexts to target BC, from a novel point of view, providing a suggestion of possible orchestrated therapeutic strategies to confront the immunosuppressive nature of breast tumors. Table 2 presents some therapeutics that target the immunosuppressive effects of the TME in BC through *in vivo* or *in vitro* studies. It also summarizes the effect of the agents on the profile of T cell polarization, miRNAs, and signaling pathway components, particularly those addressed previously in this review.

**Table 2:** Clinical trials and preclinical studies on the effect of secretome inhibitors and/or immunotherapy on BC, T cell polarization, and microRNA profiles

Therapeutic classification		Agent	Agent type	Target	NCT or *in vivo* study	Type of BC/cell line	Agent effect on T cells	Agent’s effect on miRNA profile	Agent’s effect on signaling pathway/TF^b^	References
Secretome inhibitors	cytokines and cytokine receptors, inhibitors	galunisertib (LY2157299)	small molecule	TGF-βR1	NCT02672475 (phase I)	metastatic androgen receptor negative triple-negative BC	Tregs ↓ IL-17-producing CD4^+^ cells ↓	miR-21 ↓	–	Hira et al. and Qu et al.^198^^,^^199^
		fresolimumab	mAb^a^	TGF-β	NCT01401062 (phase II)	metastatic BC	Treg ↑ CD8^+^ memory ↑ MDSCs ↓	–	–	Formenti et al.^200^
		daclizumab	mAb	CD25(IL-2Ra)	NCT00573495 (phase I)	advanced BC	Treg ↓ Th17-DP^c^ ↓ CD8^+^ T cells ↑	–	FOXP3 ↓	Rech et al., Yu et al., and Sanhuez et al.^201^^,^^202^^,^^203^
		denileukin diftitox (ONTAK)	recombinant cytotoxic fusion protein	CD25(IL-2Ra)	NCT00425672 (phases I and II)	advanced BC	Treg ↓	–	–	Morse et al.^204^
Secretome inhibitors (continued)	cytokines and cytokine receptors, inhibitors (continued)	basiliximab	mAb	CD25(IL-2Ra)	NCT01660529 (Early phase I)	metastatic BC	Treg ↓	–	FOXP3 ↓ IL-10 secretion ↓	López-Abente et al.^205^
		tocilizumab	mAb	IL-6R	NCT04871854 (phase II)	BC	Treg ↑ Th17↓	–	–	Samson et al. and Pesce et al.^206^^,^^207^
		sarilumab	mAb	IL-6R	NCT04333706 (phases I and II)	triple-negative BC	–	–	–	
		siltuximab	mAb	IL-6	no clinical trial yet/Tumor regression in MCF-7 xenograft murine model	MCF-7 xenograft murine model	–	–	–	Casneuf et al.^208^
	extracellular vesicles, inhibitors	imipramine	small molecule	acid sphingomyelinase	NCT03122444 (early phase I)	triple-negative BC	Treg ↑ Th17 ↓		STAT3 ↓ Akt/mTOR↓	Hannafon et al. and Seo et al.^209^^,^^210^
		Y27632	small molecule	ROCK1 and ROCK2	no clinical trial yet/tumor regression in *in vivo* and *in vitro* study in BC model	MCF-7 cell line and BC metastasis to human bone mouse model	Treg ↑ Th17 ↓	miR-17-92 ↓	IL-17A/-21 ↓ Th1- and Th2-related cytokines ↓	Catalano et al., Bai et al., Grosse et al., Nataraj et al., and Liu et al.^211^^,^^212^^,^^213^^,^^214^^,^^215^
		simvastatin	small molecule	HMG-CoA reductase	NCT03324425 (phase II)	metastatic BC	Treg ↑ Th1↓ Th17 ↓	–	IL-6, IL-12, PI3K/Akt, MAPK/Erk ↓ TGF-β and PTEN ↑	Wang et al. and de Oliveira et al.^216^^,^^217^
					NCT04418089 (phase II)	locally advanced BC				
					NCT03454529 (phase II)	stage I–II BC				
					NCT03971019 (phase III)	invasive BC				
		calpeptin	chemical compound	calpain	no clinical trial yet/suppressed MCF-7 and MDA-MB-231 cells their growth	MCF-7 and MDA-MB-231 cell lines	Th1↓ Th17↓ Th2 ↑	–	IL-2 ↑ IFN-γ and IL-17a ↓	Trager et al. and Mataga et al.^218^^,^^219^
		cannabidiol	small molecule	cannabinoid receptors	NCT05016349 (phase III)	early BC	Treg ↑ Th1↓ Th17↓	miRNA-21 ↓ miRNA-146a ↓ miRNA-155 ↓	mTOR and Akt ↓	Mataga et al., Zhang et al., Dhital et al., Baban et al., Al-Ghezi et al., and Kosgodage et al.^219^^,^^220^^,^^221^^,^^222^^,^^223^^,^^224^
					NCT04754399 (phase II)	BC				
					NCT04398446 (phase II)	non-metastatic breast				
					suppressed MDA-MB-231 cells growth	MDA-MB-231 cell lines				
		GW4869	small molecule	–	no clinical trial yet/reduced exosome production *in vitro* models of BC	MDA-MB-231 human BC cells and 4T1 mouse mammary tumor cells	Treg ↓ CD8^+^ ↑	–	–	Chen et al. and Donate et al.^225^^,^^226^
	indoleamine 2,3-dioxygenase (IDO) inhibitors	indoximod (1-methyl-D-tryptophan)	small molecule	IDO	NCT01792050 (phase II)	metastatic BC	Treg ↓ IL-17-producing helper T cells ↑ CD8^+^ T cells ↑	–	FOXP3 ↓	Brincks et al.^227^
					NCT01042535 (phases I and II)	metastatic BC				
		epacadostat	small molecule	IDO	NCT03328026 (phase I and II)	metastatic or locally recurrent BC	Treg ↓ CD8^+^ ↑	–	–	Jochems et al.^228^
		navoximod (GDC-0919)	small molecule	IDO	NCT02471846 (phase I)	triple-negative BC	Treg ↓ CD8^+^ ↑	–	–	Ge et al. and Nayak-Kapoor et al.^229^^,^^230^
Transcription factor and signaling pathway inhibitors	FOXP3 inhibitor	AZD8701	antisense oligonucleotide	FOXP3	NCT04504669 (phase I)	triple-negative BC	Treg ↓	–	FOXP3 ↓ CTLA-4 ↓ GITR ↓	Sinclair et al.^231^
	RORγt inhibitors	digoxin	small molecule	reduces RORγt	NCT01763931 (phase II)	newly diagnosed operable BC	Th17 ↓	–	HIF-1α ↓ RORγt ↓ IL-6-mediated Treg-to-Th17 cell conversion ↓	Capone et al., Wang et al., Huh et al., and Semenza et al.^232^^,^^233^^,^^234^^,^^235^
		ursolic acid	small molecule	reduces RORγt	no clinical trial yet/breast tumor regression in *in vivo* and *in vitro* models	several cell lines such as MCF-7/MDA-MB-231 and xenograft models	Treg ↓ Th17 ↓ MDSCs ↓	–	IL-17A ↓ STAT5 ↓ IL-10 ↓	Iqbal et al. and Zhang et al.^236^^,^^237^
	STAT3 inhibitor	pyrimethamine	small molecule	STAT3	no clinical trial yet/breast tumor regression in *in vivo* and *in vitro* models	TUBO and TM40D-MBcell lines, Tumor-bearing mice	CD8^+^ T cell ↑	–	STAT3 ↓	Khan et al.^238^
	JAK1/2 inhibitor	ruxolitinib	small molecule	JAK1/2	NCT02876302 (phase II)	triple-negative inflammatory BC	Treg ↑ Th17↓	–	–	Li et al. and Hosseini et al.^239^^,^^240^
		tofacitinib	small molecule	JAK	no clinical trial yet/Tofacitinib boosts the anti-cancer activity of immunotoxins *in vivo*	mice bearing MDA-MB-468, triple-negative BC xenografts	Th17↓	–	IL-21 ↓ IL-22 ↓ IL-23R ↓ STAT3 ↓	Fasching et al., Ghoreschi et al., and Simon et al.^241^^,^^242^^,^^243^
	Calcineurin inhibitor	tacrolimus	small molecule	calcineurin	NCT04541290 (phases I and II)	women with stage 1–2 lymphedema due to BC treatment	Treg ↓ Th17↓ Tfh (follicular T helper cell) ↓	–	NFATs ↓	Siamakpour-Reihani et al. and Li et al.^244^^,^^245^
Transcription factor and signaling pathway inhibitors (continued)					NCT00109993 (phase II)	BC				
					NCT04390685 (phases I and II)	prevention of BC-related lymphedema with tacrolimus				
	PD-1/PDL-1 inhibitors	nivolumab	mAb	PD-1	NCT03789110 (phase II)	HER2-negative BC	Treg ↓ CD8^+^ T cell ↑	–	–	Capasso et al.^246^
		pembrolizumab	mAb	PD-1	NCT01848834 (phase I)	advanced triple-negative BC	Treg ↓	–	mTOR ↑ STAT1 ↑ MAPK ↓	Sasidharan Nair et al. and Nanda et al.^247^^,^^248^
		durvalumab	mAb	PD-L1	NCT02802098 (Early phase I)	HER2-negative advanced BC	Treg ↓ CD8^+^ T cell ↑	–	MAPK ↓	Quintela-Fandino et al.^249^
					NCT02685059 (phase II)	triple-negative BC				
					NCT02628132 (phase I and II)	metastatic triple-negative BC				
	CTLA-4 inhibitors	ipilimumab	mAb	CTLA-4	NCT01502592 (phase I)	early-stage/respectable BC	Treg ↓ CD8^+^ T cell ↑	–	–	McArthur et al.^250^
					NCT03546686 (phase II)	triple-negative BC				
					NCT03818685 (phase II)	triple-negative BC				
					NCT03650894 (phase II)	HER2-negative BC				
					NCT03409198 (phase II)	BC				
Treg inhibition	chemotherapeutic agents with Treg-lowering effects	mitoxantrone	small molecule	off-target Treg reduction	NCT00002544 (phase III)	metastatic BC	Treg ↓ Th1 ↓ Th17 ↓	–	IL-23 ↓ HIF-1α ↓	Verma et al., Barni et al., and Burns et al.^251^^,^^252^^,^^253^
					NCT04927481 (phase II)	HER2 negative BC				
		imatinib	small molecule	off-target Treg reduction	NCT00193180 (phase II)	metastatic BC	Treg ↓ Th17 ↓	–	STAT3 ↓ STAT5 ↓	Larmonier et al. and Chen et al.^254^^,^^255^
		dasatinib	small molecule	off-target Treg reduction	NCT00546104 (phase II)	advanced BC	Treg ↓ Th17 ↓ Th1 ↑ CD8^+^ T cell ↑	–	JAK/STAT pathway ↓ STAT5 ↓	Wei et al. and Chen et al.^256^^,^^257^
		fludarabine	small molecule	off-target Treg reduction	NCT03635632 (phase I)	BC	Treg ↓ Th17 ↓	–	STAT1 signaling ↓	Beyer et al. and Hus et al.^258^^,^^259^
		paclitaxel	small molecule	off-target Treg reduction	NCT03959397 (phase II)	BC	Treg ↓	–	PI3K/AKT signaling ↓	Emens et al. and Li et al.^260^^,^^261^
		cyclophosphamide	small molecule	off-target Treg reduction	NCT00635050 (phase II)	locally advanced BC	Treg ↓	–	PI3K/Akt/mTOR signaling ↓	Zhu et al.^194^

a mAb, monoclonal antibody.

b TF, transcription factor.

c Th17-DP, a subtype of Th17 that produces very low levels of IL-2 constitutively.

### Targeting cytokines, cytokine receptors, and transcription factors

**Figure 3:**
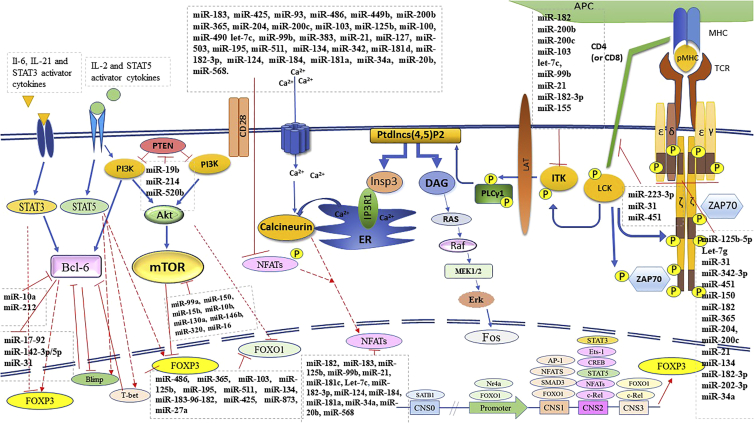
MicroRNAs targeting proteins in different pathways, which are activated in CD4^+^ effector T cells and deviate T cell fate toward FOXP3^+^ T cells

TGF-β/TGF-βR, IL-2/IL-2Ra (CD25), and IL-6 are among important cytokines and cytokine receptors in Treg/IL-17-producing, Th17 cell function, and targeting these molecules has been a subject for preclinical and/or clinical studies.^9^ TGF-β suppresses effector T cells and induces angiogenesis in tumors. Thus, TGF-β blockade may help immune responses and inhibit tumor growth.^200^ Galunisertib (LY2157299) is an inhibitor of TGF-β receptor-I activation that limits Tregs and IL-17-producing Tregs.^198^ In an active phase I clinical trial, galunisertib and paclitaxel are being tested in metastatic triple-negative BC patients (NCT02672475). Fresolimumab is an antibody against TGF-β, in a clinical trial (NCT01401062) with administration in high doses combined with radiation. It induces a boost in the CD8^+^ memory pool and a decrease in MDSCs. Although metastatic BC patients who received a high dose of fresolimumab plus radiotherapy showed better survival rates and favorable immune response, circulating Tregs increased. This study showed that targeting TGF-β alone is not sufficient in controlling tumor growth.^200^ Several Smad proteins play a crucial role in TGF-β signaling and in controlling Treg or Th17 cell differentiation.^64^^,^^127^ Smad-7 represses Smad-2/3/4 complex formation by targeting Smad-2 or Smad-3 and activates transcriptional responses of TGF-β.^64^^,^^127^ Despite the role of miR-21 in Treg stability, besides miR-181c, miR-21 enhances the differentiation of IL-17-producing T cells by targeting Smad-7, resulting in elevation of the levels of Smad-2/3. To compensate FOXP3 expression associated with miR-21 deficient in iTreg differentiation and stability, higher levels of IL-2 can be impressive and prolong iTreg stability.^41^ However, IL-2 levels vary in the different conditions associated with BC patients (Figure 3).^29^

IL-2/IL-2Ra (CD25) can be targeted with a number of monoclonal antibodies (mAbs).^262^ Daclizumab is an anti-CD25 mAb that induced a prolonged decrease in Tregs and an increase in CD8^+^ T cells after administration to metastatic BC patients with an experimental cancer vaccine. In this study, the boosting of effector T cells did not cause an autoimmune response and Treg reprogramming, with a loss in its suppressive function and FOXP3 expression shown *in vitro*.^201^ Daclizumab also targeted the expansion of Th17-DP, which is a Th17 subtype that constitutively produces very low levels of IL-2 and ameliorated its inflammatory effects.^202^ In another phase I clinical trial (NCT00573495), daclizumab and prevnar in combination with hTERT/Survivin and telomerase peptide vaccination were administered for advanced BC treatment.^203^ Denileukin diftitox (ONTAK) is a fusion between IL-2 with the active domain of diphtheria toxin that can neutralize cells with high expression of CD25.^204^ A decrease in Tregs was achieved through repeated dosing of denileukin diftitox in clinical studies on breast and colon cancer patients and in a preclinical study on donor PBMCs, and patients treated with Treg reduction showed a better specific immune response to tumor antigen.^204^ Another mAb that targets IL-2Ra is basiliximab, one of the agents used in a completed early phase I clinical trial (NCT01660529). Basiliximab inhibited Treg proliferation, FOXP3 expression, and IL-10 secretion capability, lowering Treg numbers and function.^205^

Pyrimethamine, a STAT3 inhibitor, has anti-cancer and immune-stimulatory actions in a BC murine model.^238^ Ruxolitinib, a JAK1/2 inhibitor that decreased Th17 cells and increased Tregs, has been tested in a completed phase II clinical trial (NCT02876302) in inflammatory BC with paclitaxel, doxorubicin, and cyclophosphamide.^239^^,^^240^ Also, ruxolitinib decreased breast tumor invasiveness in a xenograft BC model.^263^ Tofacitinib is a small molecule that inhibits JAK protein and abolishes Th17 cell generation by curbing IL-21, IL-22, and IL-23R expression.^241^^,^^242^ In a BC xenograft model, this agent altered the TME by suppression of the chemokines that attracted inflammatory cells.^243^ Digoxin and ursolic acid are among RORγt inhibitors.^232^^,^^236^ Digoxin, which suppresses HIF-1α and its downstream targets, including RORγt, reduces Th17 cells and inhibits IL-6-mediated Treg-to-Th17 cell conversion.^232^^,^^233^^,^^234^^,^^235^ Digoxin usage has been investigated as a drug for BC patients in a phase II clinical trial (NCT01763931) after showing preclinical efficacy.^232^ Some chemotherapeutic agents can have off-target immunotherapy effects, including changes in the level of immune cells such as Tregs, as well as their original anti-cancer mechanisms.^251^^,^^260^ In some cases of BC therapy, along with the proper agents for specific targeting in cancer therapy, administration of a proper dose and sequence of a chemotherapeutic drug can suppress the immune evasion and tumor-specific immune tolerance.^264^

Mitoxantrone, imatinib, dasatinib, fludarabine, paclitaxel, and cyclophosphamide are among these agents.^265^ Mitoxantrone, a DNA binding agent that binds to deoxyribose on DNA to cause strand breakup and unraveling, significantly lowered Tregs when administered to patients with BC.^251^^,^^252^ Mitoxantrone also has diminishing effects on the levels of Th1 and Th17 cells by IL-23 suppression.^253^ In a completed phase III clinical trial (NCT00002544) and a recruiting clinical trial (NCT04927481), mitoxantrone hydrochloride has been tested on women with metastatic BC and patients with advanced HER2-negative BC, respectively. Two tyrosine kinase inhibitors, imatinib and dasatinib, impair Treg function^254^^,^^256^ as well as Th17.^255^^,^^257^ Imatinib is also effective in the reduction of STAT3 and STAT5 activation in Tregs.^254^ After dasatinib treatment, significantly increased levels of CD8^+^ T cell levels were revealed compared with before treatment.^256^ Fludarabine, a purine analog used as a chemotherapy agent,^266^ has been used as one of the agents in several BCs, including recruiting clinical trials (NCT05142475, NCT04639245, NCT01174121, and NCT04102436) and completed clinical trials (NCT01105650, NCT01967823, NCT02111850, and NCT00365287). Fludarabine in these clinical trials was part of a chemotherapy regimen alongside the main drug. In one of these clinical trials (NCT02111850), patients received a cyclophosphamide plus fludarabine regimen and a lymphodepleting regimen that targeted MAGE-A3 TCR for patients with metastatic BC, cervical cancer, renal cancer, urothelial cancer, and melanoma. The results for of one of the patients with cervical cancer showed 85% shrinkage of the tumor by 8 months.^267^

Treatment of chronic myeloid leukemia patients with fludarabine showed decreased Treg and Th17 cell frequency.^258^^,^^259^ In MCF-7 cells, miRNAs-21/-222/148a might be involved in fludarabine resistance.^268^ Two other chemotherapy drugs, cyclophosphamide and paclitaxel, also affect Treg depletion,^194^^,^^260^ and both these agents inhibit the activation of PI3K/Akt signaling pathways.^261^ Moreover, in a phase II clinical trial for metastatic BC, using both cyclophosphamide and paclitaxel has shown good efficacy.^269^ Tacrolimus, an immunosuppressive agent, is an inhibitor of calcineurin that results in suppression of NFAT nuclear translocation. Th17, Treg, and follicular Th cells are all suppressed by tacrolimus. This agent has been investigated in several preclinical and clinical (NCT04541290, NCT00109993, and NCT04390685) studies.^244^

### OncomicroRNA-based therapies

Recently, oligonucleotides have been suggested to correct the gene expression imbalances and related cellular pathways in cancer.^270^ In this regard, miRNA modulators (mimics and antagonists) have emerged as new therapeutic strategies for the treatment of cancer.^271^ There are two main miRNA-based therapeutics: miRNA mimics to restore tumor-suppressor miRNAs and miRNA-inhibiting molecules such as antisense oligonucleotides (ASOs), also known as antagomirs, and miRNA sponges to suppress oncogenic miRNAs.^271^ Targeting overexpressed miRNAs in cancer is a strategy that can restore the level of their target genes.^272^ There are several clinical studies of BC that use the expression levels of specific miRNAs as diagnostic tests (NCT04720508, NCT04778202, NCT03779022, NCT04771871, NCT04906330, and NCT01231386). Here, however, we only discuss the therapeutic potential of targeting some of the most important miRNAs that have been discussed in previous parts of this review. One of the strategies to target oncogenic miRNAs with similar activity, for example, members of the miR-182/-183/-96 cluster, is simultaneous inhibition of all three at once.^273^ Targeting all three miRNAs together with an ASO showed better anti-proliferative effects on tumor cells than knockdown of each miRNA alone and miRNAs-182 and -96 compensated for the miR-183 knockdown effects in BC cells, in both MCF-7 and T47D cells. Antagomir-treated cells which had received miR-96 and miR-182 antagomirs showed significantly reduced cell growth, but this reduction in cell proliferation was not significant after receiving miR-183 antagomir. The reason may be that, in the miR-182/-183/-96 cluster, after knockdown of the miR-183, the other two miRs (miR-96 and -182) had compensatory effects for the miR-183. Therefore, it is best to knock down all these cluster members together to obtain the best result.^181^^,^^273^ Antagonizing miRNA-155 activity with an miRNA sponge in an *in vivo* study with mouse leukemia cells and human melanoma cells potentiated P53 and SOCS1 induction. This study showed that it was possible to reactivate cytokine signaling by targeting oncogenic miRNAs such as miRNA-155 and miR-19.^274^ Targeting miRNA-155 with a locked nucleic acid-modified oligonucleotide, named cobomarsen (MRG-106), inhibited JAK/STAT/MAPK/ERK and PI3K/Akt signaling pathways and activated apoptosis in mycosis fungoides and human lymphotropic virus type 1 cutaneous T cell lymphoma cell lines.^275^ To target miRNA-125b, extracellular vesicles loaded with miRNA-125b-ASO delivered effective suppression results, and significantly reduced tumor growth in a nasopharyngeal mouse model using an antagomir for miR-125b.^276^^,^^277^

Using anti-miRNA-214 oligonucleotides R97/R98 causes a reduction in tumor progression in multiple mouse models, including mice with BC.^278^ In this study, it was revealed that adeno-associated viruses-serotype 8 (AAV8) expressing miRNA-214 sponges had the same function as R97/R98 on the tumors.^278^ In BC, targeting miRNA-214 significantly reduced the levels of PI3K/Akt/mTOR.^279^ The inhibitors of miRNAs-221/-222 in colorectal cancer reduced cell growth and colony formation and STAT3 and IL-6 activity.^117^ PTEN was elevated after suppressing the expression of miRNAs-221/-222 with a miRNA sponge in oral squamous cell carcinoma cells.^280^ In a triple-negative BC (TNBC) mouse model, self-assembled RNA-triple-helix hydrogel nanoconjugates and scaffold were used to deliver miRNA-221 antagomir. The results showed 90% tumor shrinkage after treatment.^281^ Locked nucleic acid (LNA)-anti-miR-21 downregulated miRNA-21 and induced cell death in colorectal adenocarcinoma, melanoma, and glioblastoma cell lines.^282^^,^^283^ In the last example on glioblastoma cell lines, PTEN expression was increased after treatment with LNA-anti-miR-21.^283^ Moreover, miRNA-21 expression level is going to be measured as a diagnostic test in one clinical trial (NCT05151224) in patients with BC, before and after neoadjuvant systemic therapy. In the microenvironment of different BC subtypes, Tregs play a crucial role in the development of the disease, and the Treg prognostic effect correlates with tumor stage and molecular subtype.^284^^,^^285^ FOXP3 knockdown with AZD8701, an antisense oligonucleotide, has exhibited a significant tumor growth reduction in an *in vivo* model.^231^ Treg-suppressive function was diminished in *in vitro* FOXP3 inhibition assay with AZD8701. Also, FOXP3 knockdown generated a humanized mouse model. FOXP3 suppression with AZD8701 in primary human Tregs also reduced the expression level of FOXP3 target genes, including CTLA-4, ICOS, CCR8, and GITR, by 25%–50%. In another *in vivo* and *in vitro* preclinical study, FOXP3 silencing with a chimeric aptamer limited the immunosuppressive function of Tregs.^286^

### Extracellular vesicles

Breast-tumor-derived exosomes can shuttle malignancy inducer and immunosuppressive proteins and miRNAs in the plasma of BC patients.^209^ Exosome generation inhibitors (imipramine, Y27632, simvastatin), as well as exosome secretion inhibitors (calpeptin, cannabidiol, GW4869), have been used in clinical or *in vivo* studies in malignancies.^211^^,^^220^ Imipramine has inhibitory activity on acid sphingomyelinase that is involved in catalyzing sphingomyelin hydrolysis to ceramide, and this process involves extracellular vesicle formation and release.^211^ Studies revealed that imipramine, despite that it reduces Th17 generation as well as inhibits STAT3 and Akt/mTOR, significantly increases Tregs.^212^^,^^213^ Y27632 as a competitive inhibitor of both Rho-associated protein kinase ROCK1 and ROCK2 significantly increases Tregs.^211^^,^^214^ In addition, despite simvastatin and cannabidiol exerting their roles as suppressive agents for Th17, they induce Tregs.^216^^,^^217^^,^^221^^,^^222^^,^^223^ Hence, imipramine, Y27632, and cannabidiol, despite their benefits, may represent adverse effects by increasing Tregs in the treatment of BC. GW4869, another inhibitor of exosome release, reduced exosome production in *in vitro* BC models.^287^ After treatment with GW4869, the inhibitory effect of Tregs was reduced as the protein levels of CD8^+^ T cells returned to the normal state (up to 90%). This study also showed that the inhibitory effect of Tregs on CD8^+^ T cells was dependent on the exosomes secreted by Tregs.^225^ GW4869 also reduced the release of extracellular vesicles from Th17 cells.^226^ Therefore, GW4869 could be a suitable agent for BC therapy by targeting both Th17 and Treg cells.

### Indoleamine 2,3-dioxygenase

IDO is a potent factor in BC progression and tumor tolerance.^197^ Tumor cells produce IDO to turn tryptophan into kynurenine, which interacts with aryl hydrocarbon receptor in the TME, thereby leading to tryptophan depletion and facilitating Treg production.^288^ Since IDO suppresses T cell proliferation in the TME, it can be a potential target to conquer BC.^197^ One of the inhibitors of IDO is indoximod (1-methyl-D-tryptophan), which has been part of therapeutic regimens for BC (NCT01792050, NCT01042535, NCT02913430). Indoximod promotes the development of IL-17-producing helper T cells and CD8^+^ T cells and increases RORC transcription while suppressing the differentiation of Tregs by reducing FOXP3 transcription.^227^ Epacadostat and navoximod (GDC-0919) are selective small-molecule inhibitors of IDO that had significant limiting effects on the proliferation of IDO-induced Tregs.^228^^,^^229^ When dendritic cells were treated with epacadostat, their stimulatory capacity on CD8^+^ antigen-specific T cells increased. Epacadostat is being tested in a phase I/II clinical trial on BC patients (NCT03328026). Navoximod also showed CD8^+^ T cell activating effects when combined with anti-PD-L1 and anti-OX40 in preclinical studies, and there is an ongoing phase Ib clinical trial using a combination of navoximod with atezolizumab in patients with solid tumors (NCT02471846).^230^

### Immune checkpoints

It seems that tumor cells preserve themselves against T cells using different strategies. Tumor cells target inhibitory receptors of effector and cytotoxic T cells such as PD-1 and the cytotoxic CTLA-4 by their ligand PD-L1/PD-L2 and CD80 on their surface.^159^ Interaction between CTLA-4 on T cells and CD80 on tumor cells leads to protein kinase B (Akt) pathway inhibition and IL-2 function in T cells.^6^^,^^10^ Also, the interaction between PD-1 or PD-2 on the surface of T cells and its ligand PD-L1 on the surface of tumor cells or antigen-presenting calls (APCs) suppresses ζ-chain-associated protein kinase (ZAP70) by SH2 containing protein tyrosine phosphatase-2 (SHP2). Subsequently it attenuates all three main signal transduction pathways related to TCR interaction with APCs, including MAPK, Insp3, and DAG.^159^^,^^160^

### Immune checkpoint inhibition in Treg/IL-17-producing Treg/Th17 cell axis

#### Targeting PD-1/PD-L1

Targeting PD-1/PD-L1 can eliminate cancer cells through activation of intratumoral immune cells that kill tumor cells.^247^ In early-stage and metastatic BC, several PD-1 and PD-L1 inhibitors are currently being investigated.^289^ In BC, an immune escape is promoted through upregulation of PD-L1 and Tregs, and expression levels of PD-L1 have been used as a tool to predict the potential of targeting PD-L1 in solid tumors.^290^ Nivolumab, an anti-PD-1 mAb, decreased the frequency of Treg population and increased the number of CD8^+^ T lymphocyte infiltration in patient-derived colorectal cancer xenografts.^246^ In several clinical trials on different subgroups of BC, nivolumab has been part of the intervention. For instance, in the active phase II clinical trial, NCT03789110, nivolumab and ipilimumab are being tested for hypermutated HER2-negative BC. Pembrolizumab, another anti-PD-1 mAb, interferes with Treg differentiation through indirect downregulation of FOXP3 mediated by activating mTOR and STAT1 and inhibiting MAPK pathways.^247^ In a completed phase I clinical trial (NCT01848834), advanced TNBC patients received pembrolizumab, and the results of this study showed evidence of acceptable safety and therapeutic activity of this mAb for the treatment of metastatic TNBC.^248^ Nevertheless, both nivolumab and ipilimumab increased the level of Th1 and Th17.^247^^,^^291^ Durvalumab blocks the binding of PD-L1 to its receptors PD-1 and CD80 in order to aid in the anti-tumor T cell response activation.^292^ After the first durvalumab dose, in HER2-negative advanced BC, CD8^+^ T cells increased compared with baseline levels. In this study, patients who received bevacizumab for 25 months and patients who experienced benefit from subsequent durvalumab displayed decreased levels of Tregs in PBMCs, and analysis of available tissue biopsy showed decreased expression levels in Treg-related genes in TME.^249^ Several clinical trials have used durvalumab as a treatment agent in different BC subtypes. In a completed phase I/II clinical trial (NCT02628132), the combination of durvalumab and paclitaxel showed promising safety and activity in metastatic TNBC.^292^ In another randomized phase II study (NCT02685059), durvalumab in addition to an anthracycline taxane-based neoadjuvant therapy in early TNBC showed increased overall survival and disease-free survival in patients with PD-L1 positive tumors. Durvalumab is a selective and high-affinity modified human immunoglobulin G1 mAb that blocks PD-L1 binding to PD-1 and CD80.^289^

The only immune checkpoint inhibition based therapy that recently received accelerated approval for high-risk stage II–III TNBC is a combination therapy with paclitaxel and carboplatin in a neoadjuvant setting followed by a single-agent adjuvant therapy. KEYNOTE-522 is a phase III placebo-controlled, double-blinded trial comparing neoadjuvant pembrolizumab, paclitaxel, and carboplatin followed by pembrolizumab with paclitaxel, carboplatin, and placebo followed by adjuvant placebo. The results showed a significant increase in complete pathological response and event-free survival; 64.8% (95% confidence interval [CI]: 59.9–69.5) in the experimental arm versus 51.2% (95% CI: 44.1–58.3) in the placebo-chemotherapy arm.^293^ Recently, a prospective phase II clinical trial (NCT04165772) was performed in which dostarlimab, an anti-PD-1 mAb, was applied every 3 weeks for 6 months in 12 patients with mismatch-repair-deficient stage II or III rectal adenocarcinoma following standard chemoradiotherapy and surgery. In this study, all 12 patients (100% of patients; 95% CI) showed a complete response and no evidence of tumor remained. However, a longer follow-up period is necessary to evaluate the response duration.^294^

#### Targeting CTLA-4

CTLA-4 is an inhibitory molecule present on T cells, and inhibiting it with immunotherapy can promote T cell activation and proliferation while also improving anti-tumor immunity.^295^ Ipilimumab, an mAb that blocks CTLA-4, has been used as the treatment intervention agent in BC patients (NCT01502592, NCT03546686, NCT03818685, NCT03650894, and NCT03409198). In one of the clinical trials with BC patients, ipilimumab treatment depleted Tregs from TME and augmented effector T cells in patients who had received both ipilimumab and cryoablation.^250^ In an *in vivo* study, ipilimumab and tremelimumab (another anti-CTLA-4 mAb) mediated intratumoral Treg depletion and CD8^+^-to-Treg ratio augmentation and promoted tumor rejection.^296^ In another *in vivo* study of a BC mouse model, treatment with anti-CTLA-4 antibody delayed tumor growth, and anti-CTLA-4 antibody plus matrix metalloproteinase inhibitor therapy reduced the percentage of Tregs, Th17 cells, and MDSCs in tumors compared with the control group.^295^

## Conclusions

The TME affects the fate of both tumor and immune cells, especially T cells, through secretome or cell-associated factors. The Treg/IL-17-producing Treg/Th17 cell axis may play a prominent role in polarization of Th1 cells and effector CD8^+^ T cells toward suppressive T cells, thereby facilitating tumor development. Although approved clinical trials demonstrated promising therapies for cancer, they may reversibly deteriorate tumor development. Cytokine levels and their effects, such as antagonistic or synergistic functions, may delineate the fate of T cells. For example, IL-2 may activate STAT5 to induce Tregs by FOXP3 expression, but IL-6 may activate STAT3 to induce Th17 cells by RORγt expression. However, the transitional state of IL-17-producing Tregs may result from changing this balance toward STAT3 phosphorylation, which increases RORγt expression while reducing FOXP3 expression. Another component of secretome includes oncomicroRNAs that may target the mRNA of various proteins involved in different signal transduction pathways, which are necessary to activate and differentiate CD4^+^ and CD8^+^ T cells. Some oncomicroRNAs originating from endogenous or exogenous sources have positive effects, whereas few of them have negative effects on transcription factors such as FOXP3, RORγt, NFAT, Bcl-6, mTOR, and PTEN as well as critical inhibitory receptors such as PD-1 and CTLA-4. Based on the Treg/IL-17-producing Treg/Th17 cell axis, novel therapeutic approaches may be suggested to manipulate TME in favor of tumor treatment. In this regard, mAbs may effectively target tumor-favoring cytokines, for example, TGF-β and IL-2. Moreover, tyrosine kinase inhibitors such as dasatinib may potentially impair Treg function while increasing CD8^+^ T cell numbers. Immunosuppressants, such as tacrolimus, may target NFAT, which is necessary for Treg differentiation. OncomicroRNA-based therapies such as ASOs, known as antagomirs, and miRNA sponges may suppress oncomicroRNAs as new therapeutic strategies for the treatment of cancer. Lastly, checkpoint inhibitors may significantly affect TME by suppressing the initiation of signaling pathways that aggravate TME. Altogether, combination therapy using different forms of the aforementioned therapies is recommended. To this end, comprehension of the roles of Treg, IL-17-producing Treg, and Th17 cell axis in the TME may open a new window toward the provision of new therapeutic approaches based on these cells for BC as well as new insights for more targeted investigations in the future.

## Data Availability

Please get in touch with the corresponding author for data requests.
